# Enhanced resistive switching phenomena using low-positive-voltage format and self-compliance IrO_*x*_/GdO_*x*_/W cross-point memories

**DOI:** 10.1186/1556-276X-9-12

**Published:** 2014-01-08

**Authors:** Debanjan Jana, Siddheswar Maikap, Amit Prakash, Yi-Yan Chen, Hsien-Chin Chiu, Jer-Ren Yang

**Affiliations:** 1 Thin Film Nano Technology Laboratory, Department of Electronic Engineering, Chang Gung University, 259 Wen-Hwa 1st Rd, Kwei-Shan, Tao-Yuan 333, Taiwan; 2 Department of Materials Science and Engineering, National Taiwan University, No. 1, Sec. 4, Roosevelt Road, Taipei 10617, Taiwan

**Keywords:** RRAM, GdO_*x*_, Self-compliance, Resistive switching

## Abstract

Enhanced resistive switching phenomena of IrO_*x*_/GdO_*x*_/W cross-point memory devices have been observed as compared to the via-hole devices. The as-deposited Gd_2_O_3_ films with a thickness of approximately 15 nm show polycrystalline that is observed using high-resolution transmission electron microscope. Via-hole memory device shows bipolar resistive switching phenomena with a large formation voltage of -6.4 V and high operation current of >1 mA, while the cross-point memory device shows also bipolar resistive switching with low-voltage format of +2 V and self-compliance operation current of <300 μA. Switching mechanism is based on the formation and rupture of conducting filament at the IrO_*x*_/GdO_*x*_ interface, owing to oxygen ion migration. The oxygen-rich GdO_*x*_ layer formation at the IrO_*x*_/GdO_*x*_ interface will also help control the resistive switching characteristics. This cross-point memory device has also Repeatable 100 DC switching cycles, narrow distribution of LRS/HRS, excellent pulse endurance of >10,000 in every cycle, and good data retention of >10^4^ s. This memory device has great potential for future nanoscale high-density non-volatile memory applications.

## Background

There is an increasing demand for next-generation high-density non-volatile memory devices because flash memories are approaching their scaling limits. Among many candidates to replace the flash memory devices, resistive random access memory (RRAM) is one of the promising candidates, owing to its simple metal-insulator-metal structure, fast switching speed, low-power operation, excellent scalability potential, and high density in crossbar structure [1-4]. Many switching materials such as TaO_*x *_[5-7], AlO_*x *_[8,9], HfO_*x *_[10-15], TiO_*x *_[16,17], NiO_*x *_[18-21], WO_*x *_[22,23], ZnO_*x *_[24,25], ZrO_*x *_[26-31], SrTiO_3 _[32,33], SiO_*x *_[34,35], and Pr_0.7_Ca_0.3_MnO_3 _[36,37] have been studied by several groups. However, the rare-earth oxide such as Gd_2_O_3_ could be a promising resistive switching material because of its high resistivity, high dielectric permittivity (*κ* = 16), moderate energy gap (*E*_g_ = approximately 5.3 eV), and higher thermodynamic stability [38]. Recently, many researchers have reported the resistive switching properties by using Gd_2_O_3_ materials [38-40]. Cao et al. [38] have reported unipolar resistive switching phenomena using Pt/Gd_2_O_3_/Pt structure with a high RESET current of 35 mA. Liu et al*. *[39] have also reported unipolar resistive switching phenomena with a high RESET current of 10 mA in Ti/Gd_2_O_3_/Pt structure. Yoon et al. [40] have reported resistive switching characteristics using MoO_*x*_/GdO_*x*_ bilayer structure with a RESET current of 300 μA. It is found that non-uniform switching and high overshoot current are the main drawbacks for practical application of non-volatile RRAM using Gd_2_O_3_ material. Even though many structures using the Gd_2_O_3_ materials have been reported, however, the cross-point memory devices using IrO_*x*_/GdO_*x*_/W structure have not yet been reported. It is reported [41] that the cross-point structure has a great potential for high-density memory application in the near future.

In this study, we discussed resistive switching phenomena of IrO_*x*_/GdO_*x*_/W cross-point memory structure. For comparison, the IrO_*x*_/GdO_*x*_/W via-hole structure has been also investigated. The IrO_*x*_/GdO_*x*_/W via-hole memory devices exhibit negative switching polarity, whereas the IrO_*x*_/GdO_*x*_/W cross-point memory devices show positive switching polarity. Switching non-uniformity and high operation voltage/current of the via-hole devices are observed. To improve the switching uniformity and control the current overshoot, we have designed the IrO_*x*_/GdO_*x*_/W cross-point memory devices. In the cross-point structure, IrO_*x*_/GdO_*x*_/W memory device shows stable and uniform positive switching due to the formation of oxygen-rich interfacial layer at the IrO_*x*_/GdO_*x*_ interface. The cross-point memory device has self-compliance bipolar resistive switching phenomena of consecutive 100 cycles with narrow distribution of high resistance state (HRS), low resistance state (LRS), good device-to-device uniformity, excellent P/E cycles of >10,000, and good data retention with resistance ratio of 100 after 10^4^ s under a low operation voltage of ±3.5 V.

## Methods

**Figure 1:**
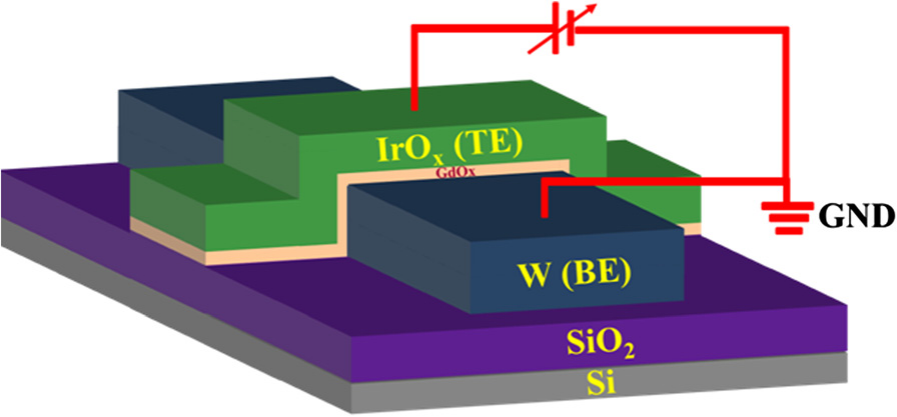
**Schematic view of IrO**_***x***_**/GdO**_***x***_**/W cross-point memory device.** Positive bias is applied at the TE, and BE was grounded during the measurement.

**Figure 2:**
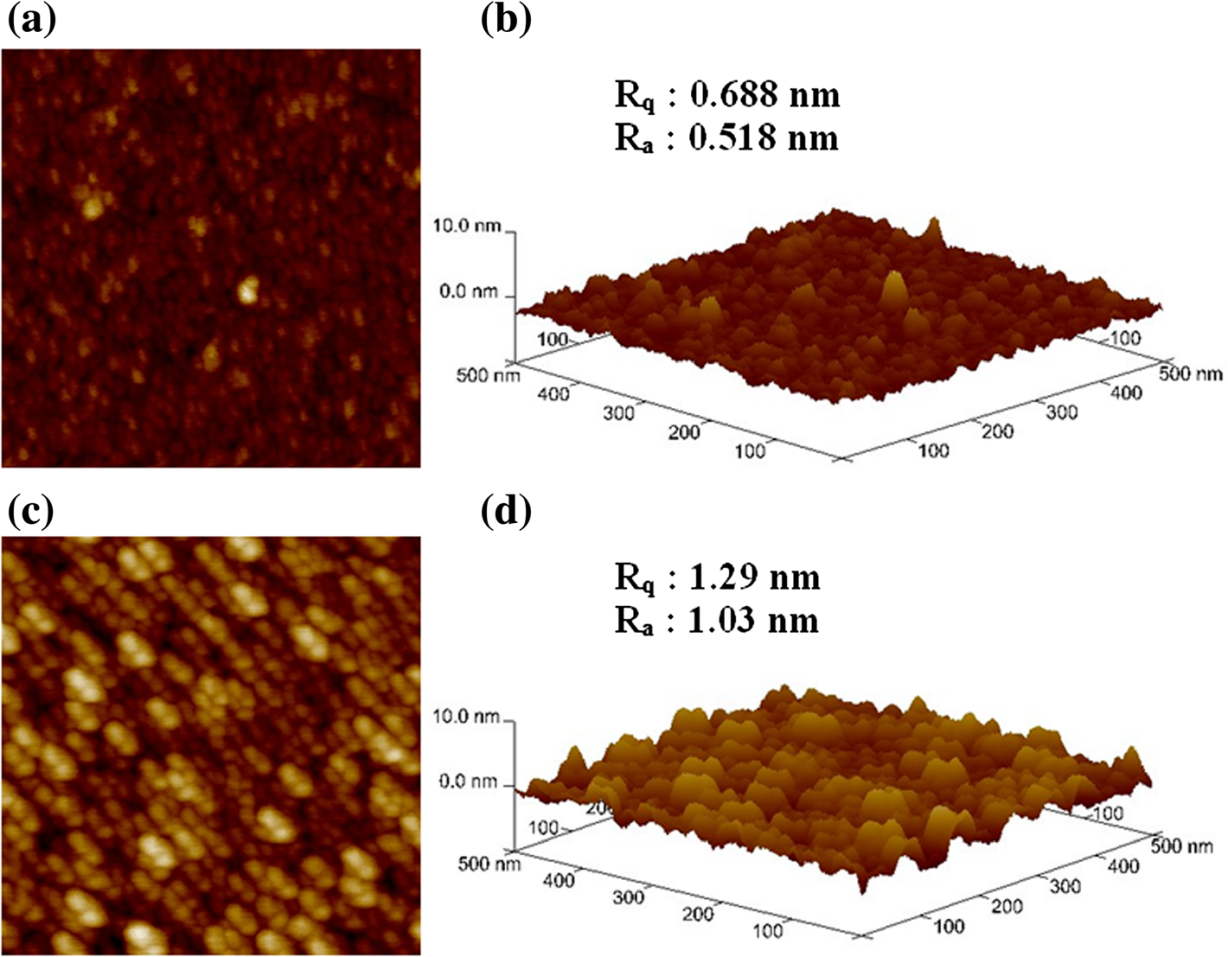
**AFM images of the films.** GdO_*x*_ film on SiO_2_/Si substrate in **(a)** 2D and **(b)** 3D views. IrO_*x*_ film on IrO_*x*_/GdO_*x*_/SiO_2_/Si stack in **(c)** 2D and **(d)** 3D views.

First, the cross-point memory devices using the IrO_*x*_/GdO_*x*_/W structure were fabricated. After conventional RCA cleaning of p-type Si wafer, 200-nm-thick SiO_2_ was grown by wet oxidation process. Then, a tungsten (W) metal layer of approximately 200 nm was deposited on the SiO_2_/Si substrate by radio frequency (rf) sputtering process. The deposition power was 150 W, and argon (Ar) with flow rate of 25 sccm was used. The W bars with different widths of 4 to 50 μm were patterned by optical lithography and wet etching process, which serve as bottom electrode (BE). Another lithography process step was used to obtain top electrode bar (TE) by lift-off. The high-*κ* Gd_2_O_3_ as a switching material was deposited by electron beam evaporation. The thickness of the Gd_2_O_3_ film was approximately 15 nm. Pure Gd_2_O_3_ shots with granules sizes of 2 to 3 mm were used. The deposition rate of Gd_2_O_3_ was 0.2 Å/s, and the power was 400 W. Then, iridium-oxide (IrO_*x*_) as a TE with a thickness of approximately 200 nm was deposited by rf sputtering. An iridium (Ir) target was used for the IrO_*x*_ TE. The ratio of Ar to O gases was 1:1 (i.e., 25/25 sccm). The deposition power and chamber pressure were 50 W and 20 mTorr, respectively. The Ir bars with different widths of 4 to 50 μm were laid 90° with W BEs. Finally, lift-off was performed to get the final devices with different sizes of 4 × 4 to 50 × 50 μm^2^. Then, the device was annealed at 400°C in N_2_ ambient for 10 min. The N_2_ pressure was 5 SLM. The cross-point memories with different arrays of 1 × 1 to 10 × 10 were designed, and the memory device at the 1 × 1 position was measured in this study. Figure 1 shows a schematic view of our IrO_*x*_/GdO_*x*_/W cross-point memory device. Figure 2 shows the topography of the Gd_2_O_3_ and IrO_*x*_ films, observed using atomic force microscope (AFM). AFM images of two-dimensional (2D) format are shown in Figure 2a,c, and three-dimensional (3D) images are shown in Figure 2b,d. The root mean square (rms, *R*_q_) and average (*R*_a_) surface roughness are found to be 0.688 and 0.518 nm of the Gd_2_O_3_ film on Si substrate, while those values are found to be 1.29 and 1.03 nm of the IrO_*x*_ film on Gd_2_O_3_/SiO_2_/Si substrate, respectively. For comparison, we have also studied the surface roughness of W BE for the via-hole and cross-point memory devices. The root mean square (*R*_q_) surface roughness of W BE for the via-hole and cross-point devices is found to be 1.35 and 4.21 nm, and the average surface roughness (*R*_a_) is found to be 1.05 and 3.35 nm, respectively [42]. It is observed that the surface roughness of W BE is higher than those of GdO_*x*_ and IrO_*x*_, which might have great impact on W BE as well as improved resistive switching characteristics.

**Figure 3:**
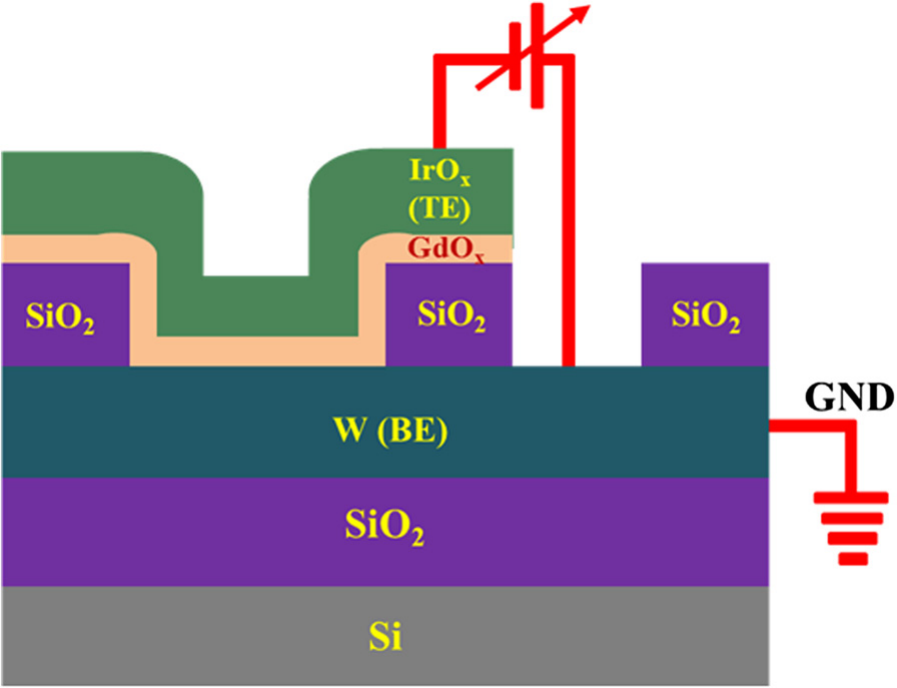
**Schematic view of resistive switching memory device in an IrO**_***x***_**/GdO**_***x***_**/W via-hole structure.** Typical device size is 2 × 2 μm^2^.

Second, the via-hole devices were fabricated for comparison. The fabrication steps are as follows. The W metal as a BE was deposited by rf sputtering on SiO_2_ (200 nm)/Si wafers. In this device, the thickness of W layer was approximately 100 nm. To form the RRAM device, the SiO_2_ layer with a thickness of approximately 150 nm was deposited. Then, a small via-hole with an active area of 2 × 2 μm^2^ was designed using standard lithography. Photoresist (PR) was used to design the pattern and was opened at the active and TE regions. Then, the Gd_2_O_3_ film with a thickness of 15 nm was deposited. Finally, lift-off was performed to get the memory device. A schematic view of our IrO_*x*_/GdO_*x*_/W via-hole structure is shown in Figure 3. During electrical measurement of the memory devices, the BE was grounded and the sweeping bias was applied on the TE.

## Results and discussion

**Figure 4:**
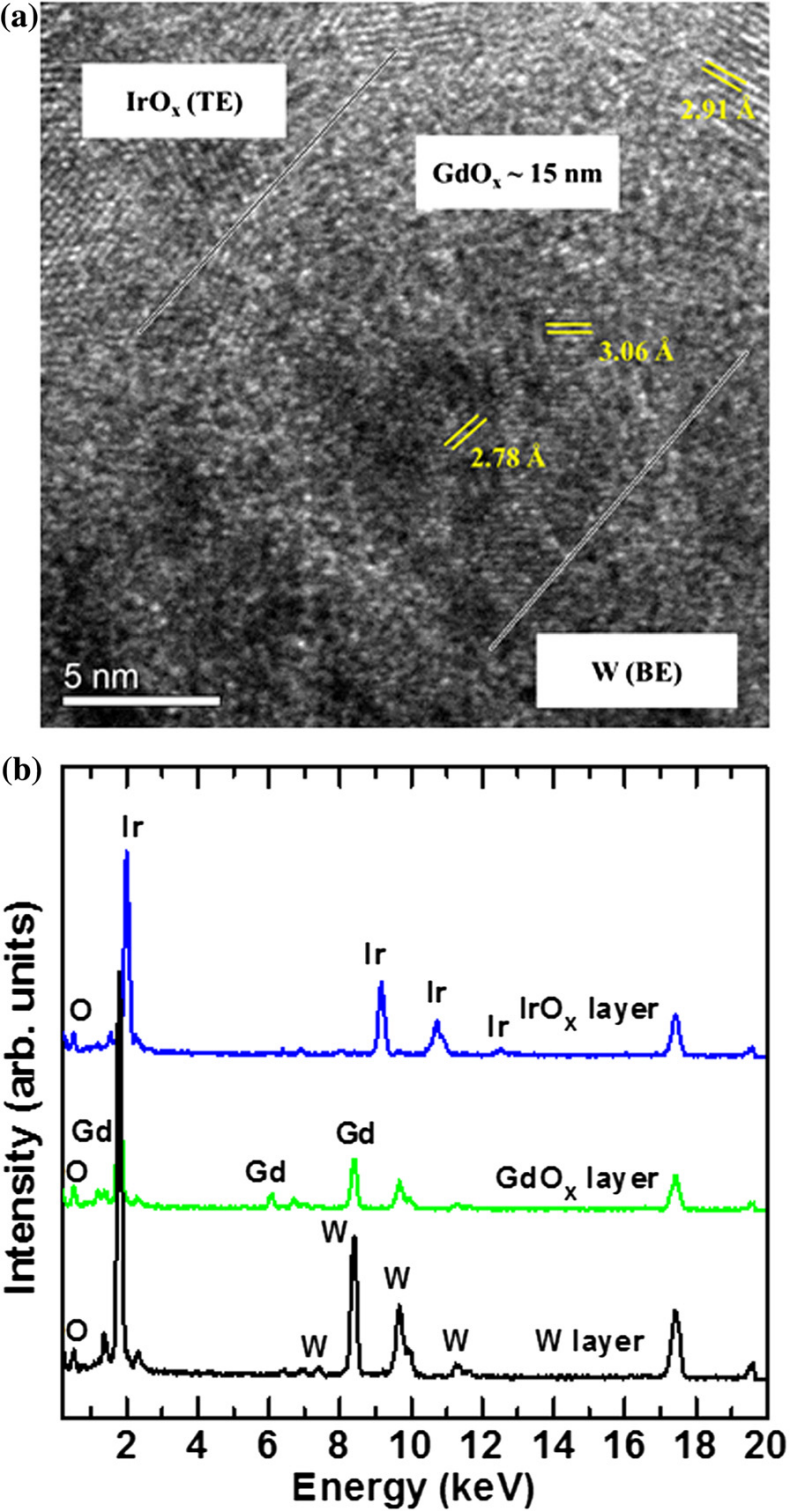
**TEM image and EDX spectra. (a)** Cross-sectional TEM image of IrO_*x*_/GdO_*x*_/W structure. Polycrystalline GdO_*x*_ film is observed. **(b)** EDX spectra show the Ir, Gd, W, and O elements.

**Figure 5:**
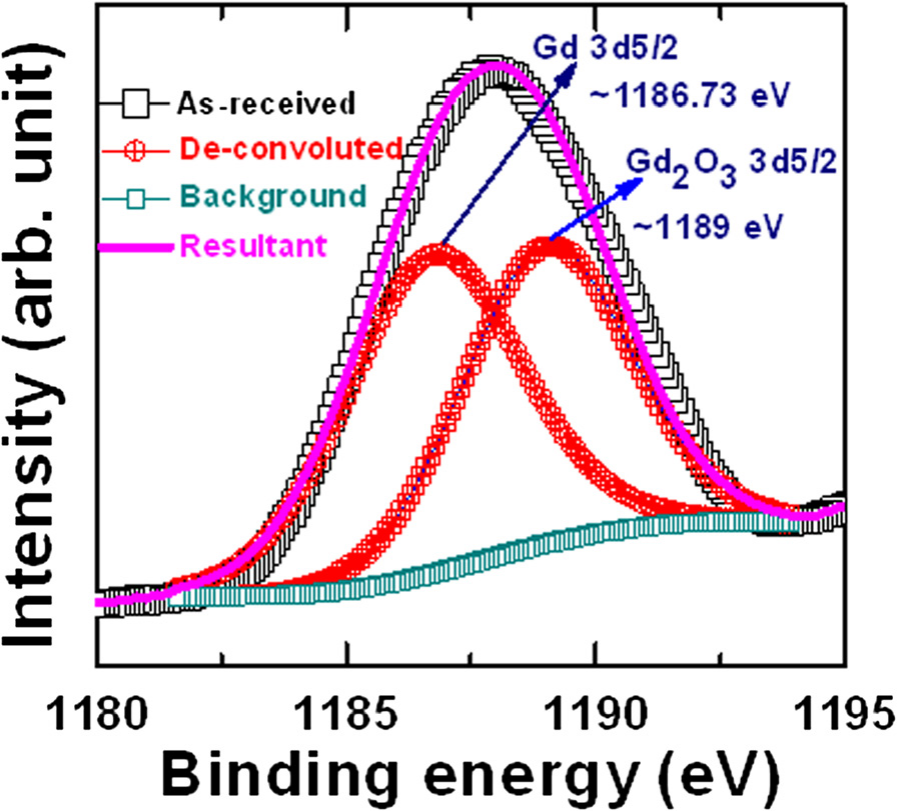
**XPS characteristics of the Gd**_**2**_**O**_**3 **_**films.** XPS spectra of the Gd 3*d* and Gd_2_O_3_ 3*d* core-level electrons.

Figure 4a shows the HRTEM image of our memory device for the as-deposited Gd_2_O_3_ film. Each layer is shown. The thickness of the GdO_*x*_ layer is approximately 15 nm. To identify the crystalline nature of the Gd_2_O_3_ film, the calculated *d* spacings are found to be 2.78 Å (101), 2.91 Å (002), and 3.06 Å (100), which are similar (2.69 Å (200), 3.09 Å (111), and 1.89 Å (220)) to those reported in the literature [43]. This suggests that this as-deposited Gd_2_O_3_ film is polycrystalline. The energy diffraction X-ray spectroscopy (EDX) spectra confirm the presence of expected elements Ir, Gd, W, and O in respective layers, as shown in Figure 4b. The X-ray photoelectron spectroscopy (XPS) spectra of Gd 3*d*_5/2_ and Gd_2_O_3_ 3*d*_5/2_ peaks are located at 1,186.73 and 1,189 eV, respectively (Figure 5), which proves a Gd-rich Gd_2_O_3_ film, i.e., GdO_*x*_. The height ratio of Gd/Gd_2_O_3_ is 1:0.93, and area ratio of Gd/Gd_2_O_3_ is 1:0.89. Arhen et al. [44] reported the same chemical bonding states at 1,186 and 1,188 eV for the Gd 3*d*_5/2_ and Gd_2_O_3_ 3*d*_5/2_ peaks, respectively. This suggests that the as-deposited Gd_2_O_3_ film is a Gd-rich GdO_*x*_ film. It is known that the grain boundary has more defects or weak Gd-O bonds. This suggests that the Gd-O bonds will break easily under external bias, and more oxygen vacancies will be created. The conducting filament will be formed through the grain boundaries. However, the nanotips on the W BE will help the structure have repeatable resistive switching memory characteristics.

**Figure 6:**
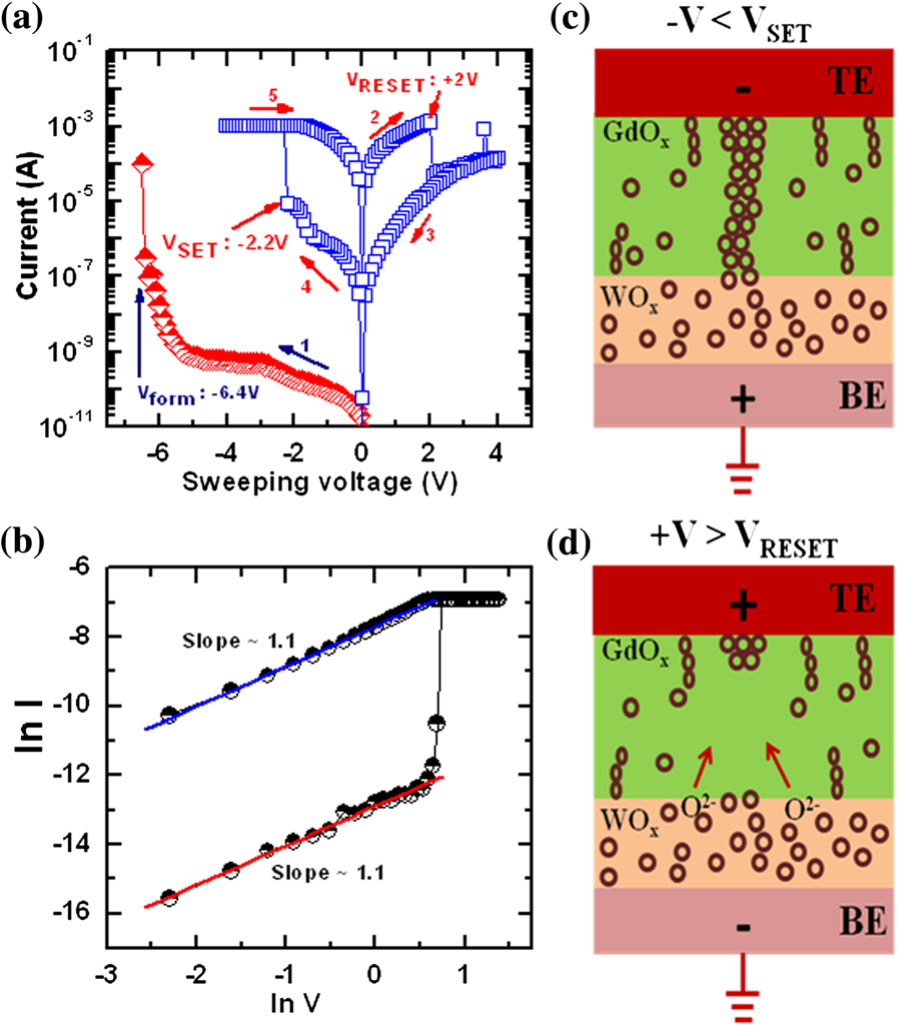
***I*-*V *switching characteristics and mechanism. (a)***I*-*V* characteristics for formation process and bipolar resistive switching characteristics of the via-hole devices, **(b)***I*-*V* fitting, **(c)** oxygen vacancy filament formation under - *V* < *V*_SET_, and **(d)** filament ruptured or oxidized under + *V* > *V*_RESET_.

Figure 6a shows the typical current–voltage (*I*-*V*) characteristics of a IrO_*x*_/GdO_*x*_/W RRAM device in via-hole structure, as illustrated schematically in Figure 3. The pristine device shows very low leakage current (arrow 1). In order to activate the resistive switching, an initial soft breakdown process (forming) was carried out by applying negative bias on the TE. The negative forming voltage (*V*_form_) is -6.4 V to initiate the resistive switching with a current compliance (CC) of 100 μA. During the formation process, the Gd-O bonds break, which creates oxygen vacancy as well as oxygen vacancy filament, and set LRS. In consequence, the oxygen ions (O^2–^) will be migrated toward the W BE and react partially at the BE. Bipolar *I*-*V* characteristics are indicated by arrows 2 to 4. The SET (*V*_SET_) and RESET voltages (*V*_RESET_) are found to be -2.2 and +2 V, respectively. To elucidate the conduction mechanism of the IrO_*x*_/GdO_*x*_/W memory device, the *I*-*V* curves are plotted in log-log scale, as shown in Figure 6b. Both LRS and HRS show ohmic conduction behaviors with a slope approximately 1.1. The LRS is ohmic because of the conducting filament formation in the GdO_*x*_ layer. The HRS is also ohmic because the electrons move through the defects of the GdO_*x*_ grain boundary. The ohmic behavior of the HRS was also reported by Jung et al. [45]. The resistive switching mechanism can be explained as follows. When negative bias is applied on the TE, the oxygen ions will move from the GdO_*x*_ layer to the WO_*x*_ layer. Then, the oxygen vacancy filament will form in the GdO_*x*_ layer, and the device switches to LRS, which is shown schematically in Figure 6c. The conducting filament will be ruptured by applying positive bias on the TE, and the device switches to HRS, as shown in Figure 6d. In this case, the O^2–^ ions will move from the WO_*x*_ layer toward the GdO_*x*_ layer and oxidize the conducting filament. Basically, the conducting filament formation/rupture is due to the oxygen ion migration. This via-hole memory device has read pulse endurance of >10^5^ cycles and good data retention at 85°C (not shown here). Both the LRS and HRS with a high resistance ratio of >10^3^ can be retained after 10^4^ s at 85°C. It is indicating that the memory device is non-volatile and stable at 85°C. However, this device operation current is high (>1 mA), and the *I*-*V* switching cycles has variation. This indicates that the via-hole device in an IrO_*x*_/GdO_*x*_/W structure needs high current operation and that multiple conducting filaments could be formed, which is difficult to control the repeatable switching, and it is also against the future application of nanoscale non-volatile memory. To resolve this issue, we have fabricated the cross-point memory device using the same IrO_*x*_/GdO_*x*_/W structure, and the improved memory characteristics are observed below.

**Figure 7:**
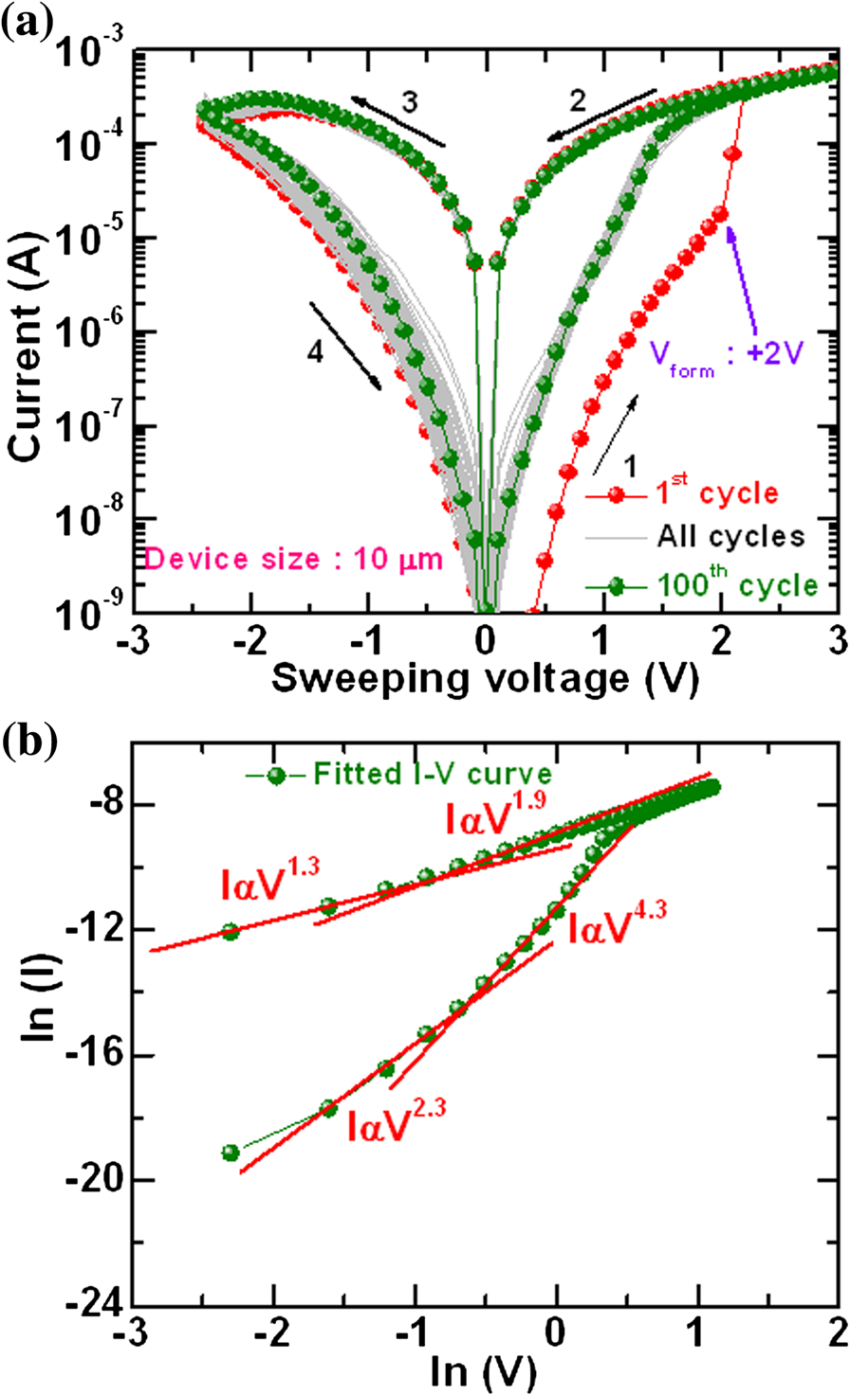
**Self-compliance *I*-*V *switching characteristics and fitting. (a)** Self-compliance Repeatable *I*-*V* hysteresis loop of our IrO_*x*_/GdO_*x*_/W cross-point memory devices. A low operation voltage of ±3 V is applied to get repeatable resistive switching characteristics. **(b)** Fitted *I*-*V* curve in a log-log scale. Both LRS and HRS show trap-controlled space charge-limited current conduction mechanism.

**Figure 8:**
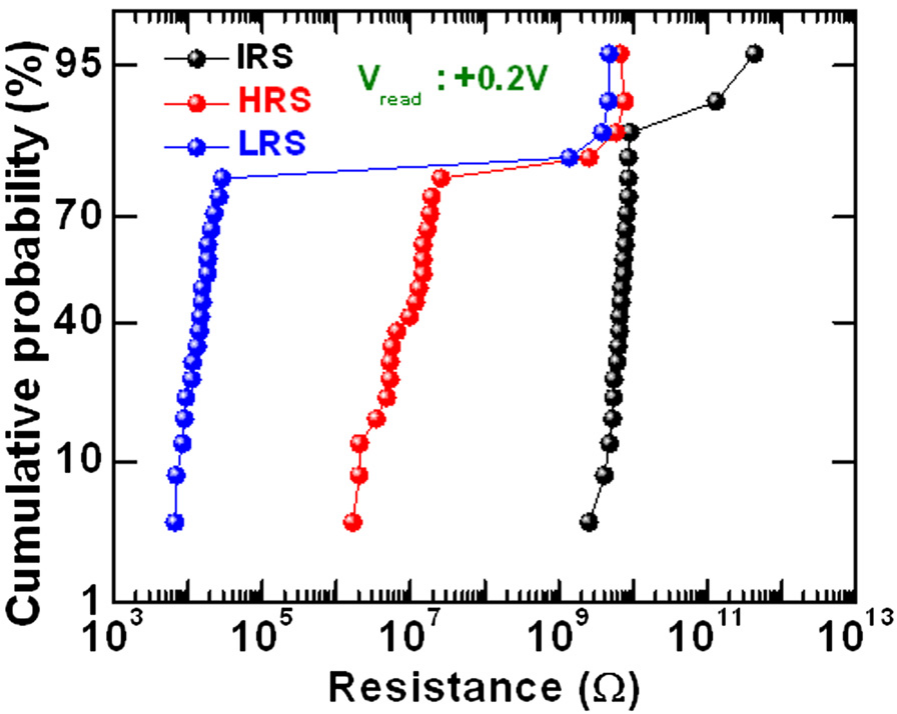
**Statistical distribution of resistances.** Statistical distribution of IRS, HRS, and LRS of the IrO_*x*_/GdO_*x*_/W cross-point memory device.

**Figure 9:**
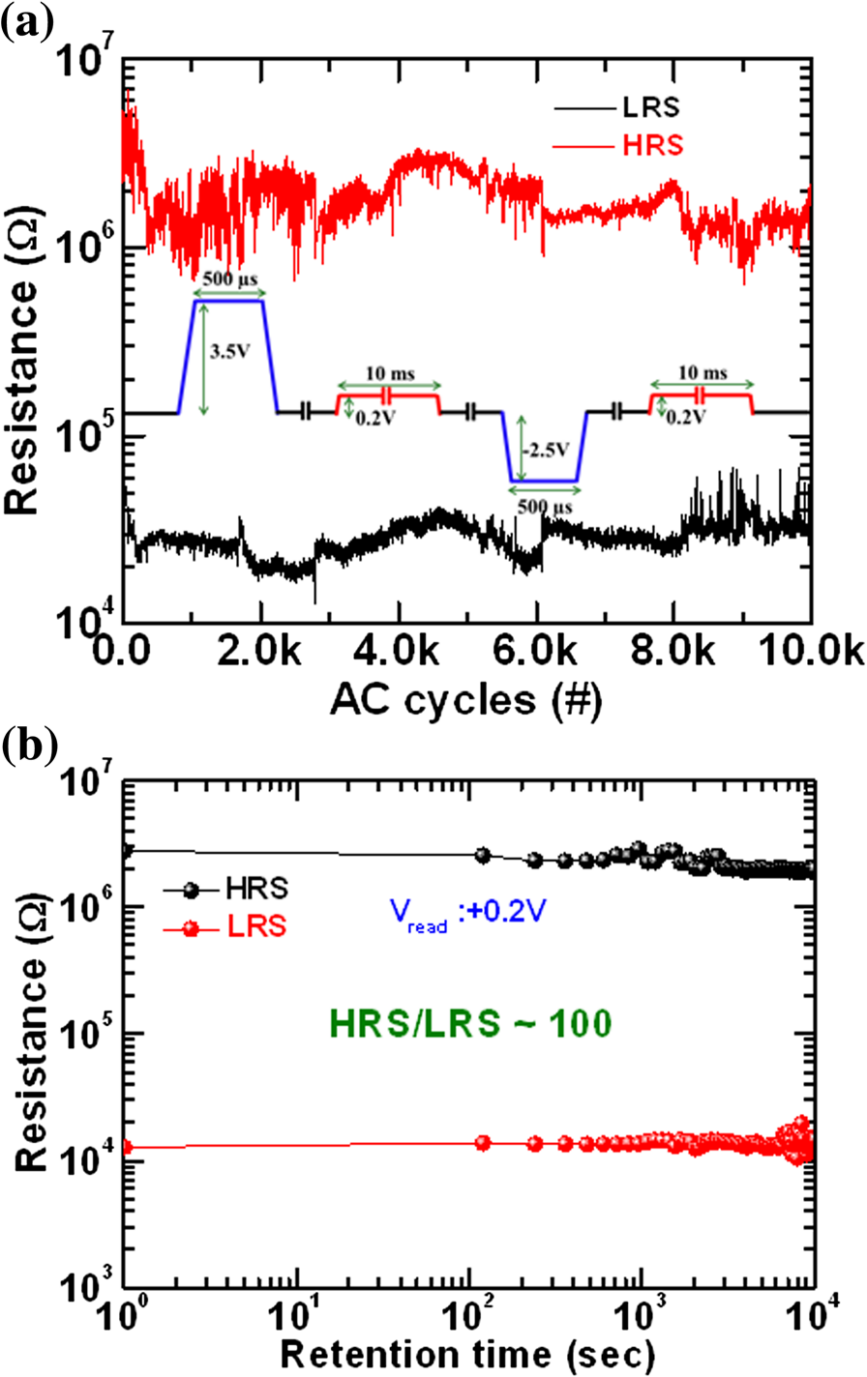
**AC endurance and data retention characteristics. (a)** Good AC endurance of more than 10,000 in every cycle of cross-point resistive switching memory device. Both resistances were read out at +0.2 V. **(b)** Good data retention characteristics of >10^4^ s is obtained.

Figure 7a shows self-compliance bipolar current–voltage characteristics of our cross-point memory device. Initially, the memory device was in HRS or initial resistance state (IRS). Therefore, the first switching cycle of the memory device shows like formation with small forming voltage (*V*_form_) +2 V, which is comparatively very lower than the via-hole device (-6.4 V) as shown in Figure 6a. This suggests that extra forming step is not required in our cross-point device if it is operated within ±3 V, which is very useful for practical realization because of its cost effectiveness and reduction of circuit complexity. The cross-point memory device exhibits Repeatable 100 cycles with small operating voltage of ±3 V, has a low-positive-voltage format, and has a self-compliance with a low current approximately 300 μA at a voltage of ±2 V. Both SET and RESET currents are almost the same, which indicates a good current clamping between the TE and BE in the switching material. To identify the current conduction mechanism, the *I*-*V* curve was fitted in the log-log scale, as shown in Figure 7b. The slope values of LRS are 1.3 (*I*α*V*^1.3^) and 1.9 (*I*α*V*^1.9^) at low- and high-voltage regions, respectively, whereas the slope values of HRS are 2.3 (*I*α*V*^2.3^) and 4.3 (*I*α*V*^4.3^) at low- and high-voltage regions, respectively. This suggests that the conduction mechanism for both LRS and HRS is trap-controlled space charge-limited current conduction mechanism (TC-SCLC). The switching mechanism is based on the formation and rupture of the conducting filament at the IrO_*x*_ (TE)/GdO_*x*_ interface, depending upon the electrical bias. By applying negative bias on the TE of the IrO_*x*_/GdO_*x*_/W via-hole devices, the O^2–^ ions drift toward the W BE and partially oxidize, as well as sink into the W BE. Due to the presence of huge numbers of oxygen vacancies into the GdO_*x*_ layer, there is much possibility to form multiple filaments resulting in non-uniform resistive switching. This phenomenon was also observed for IrO_*x*_/TaO_*x*_/W structure [46]. By applying positive bias on the IrO_*x*_/GdO_*x*_/W via-hole devices, the O^2–^ ions migrate toward the IrO_*x*_ TE. Due to the porous nature of IrO_*x*_, some O^2–^ ions drift out and some oxygen are gathered at the IrO_*x*_/GdO_*x*_ interface. The porous IrO_*x*_ film was also reported recently [47]. Oxygen-rich GdO_*x*_ layer at the GdO_*x*_/TE interface acts as a series resistance which restricts the overshoot current and makes the filament uniform. This interfacial series resistance helps achieve a repeatable switching cycle; however, few devices are controllable. On the other hand, a cross-point memory device does not exhibit switching under negative bias on the IrO_*x*_ TE, owing to higher resistivity of thinner IrO_*x*_ TE, and the device cannot reach a higher operating current. However, the cross-point memory device exhibits excellent resistive switching characteristics under positive bias on the IrO_*x*_ TE due to both the rough surface of the W BE and oxygen gathering at the IrO_*x*_/GdO_*x*_ interface. The electric field enhancement on the nanotips of the W BE and the interfacial series resistance of IrO_*x*_/GdO_*x*_ with thinner layer IrO_*x*_ TE help the structure have controllable resistive switching characteristics. Owing to the structural shape and the W BE surface differences, the cross-point memory devices have low-positive-voltage format, repeatable switching cycles, and self-compliance, and have improved switching characteristics than the via-hole devices. The similar phenomena was also reported recently [48]. However, further study is ongoing to understand the different resistive switching characteristics between the via-hole and cross-point memory devices. To check the uniformity of the cross-point memory devices, the statistical distribution of IRS, HRS, and LRS were randomly measured in more than 20 devices, as shown in Figure 8. Some devices are not switchable, which may be due to process variation from our deposition system. Most of the memory devices exhibit good distribution of IRS, HRS, and LRS. The average values (*σ*_m_) of IRS, HRS, and LRS are found to be 29.44G Ω, 9.57 MΩ, and 14.87 kΩ, and those values for standard deviation (*σ*_s_) are 89.47, 7.21, and 6.67, respectively. This suggests that the memory device has great potential for high-density memory application. Excellent program/erase (P/E) of >10,000 cycles is manifested in our IrO_*x*_/GdO_*x*_/W cross-point memory device, as shown in Figure 9a. Every cycle was measured during the measurement. The program and erase voltages were +3.5 and -2.5 V, respectively, as shown schematically in the inset of Figure 9a. After 10^4^ P/E cycles, the memory device maintain a resistance ratio of approximately 10 which is also acceptable for multilevel cell operation. Good data retention of >10^4^ s is observed, as shown in Figure 9b. Both HRS and LRS were read out at +0.2 V. A large resistance ratio of approximately 100 is maintained after 10^4^ s. This cross-point memory device paves a way in future nanoscale high-density non-volatile memory.

## Conclusions

Enhanced resistive switching characteristics using the IrO_*x*_/GdO_*x*_/W cross-point memory structure have been obtained. The HRTEM image shows a polycrystalline structure of the GdO_*x*_ films. The switching mechanism is based on the formation and rupture of the conducting filament by oxygen ion migration, and the oxygen-rich GdO_*x*_ layer formation at the IrO_*x*_/GdO_*x*_ interface acts as a series resistance to control the current overshoot effect and improves the switching uniformity as compared to the via-hole devices. The cross-point memory device shows self-compliance bipolar resistive switching phenomena of consecutive 100 cycles with narrow distribution of LRS and HRS, excellent P/E cycles of >10,000, and good data retention of >10^4^ s with resistance ratio >10^2^ under low operation voltage of ±3 V. This memory device paves a way for future nanoscale high-density non-volatile memory applications.

## Competing interests

The authors declare that they have no competing interests.

## Authors’ contributions

DJ carried out this research work, and AP helped fabricate the memory devices under the instruction of SM. YYC did TEM under the instruction of SM and JRY. HCC supported in the deposition of the Gd_2_O_3_ film. All the authors contributed to the revision of the manuscript, and they approved it for publication.

## Authors’ information

DJ is a Ph.D. student since September 2010, and AP has received his Ph.D. degree on July 2013 under the instruction of Professor SM. SM has been an Associate Professor in the Department of Electronic Engineering, Chang Gung University since August 2009. YYC is a Ph.D. student in the Department of Materials Science and Engineering, National Taiwan University, under the instruction of Professor JRY. HCC has been a Professor in the Department of Electronic Engineering, Chang Gung University since August 2010.
